# Sickle Cell Disease at a Tertiary Care Center in the Vidarbha Region of India: Protocol for a Clinical and Observational Study

**DOI:** 10.2196/80483

**Published:** 2026-01-21

**Authors:** Tanushree Budhbaware, Jaishriram Rathored

**Affiliations:** 1 Department of Central Research Laboratory and Molecular Diagnostics, Datta Meghe Institute of Higher Education and Research, Wardha, Maharashtra, 442001, India, 91 8319380593

**Keywords:** red blood cells disorder, genetic abnormality, hemoglobin S, sickle cell disease, sickle cell anaemia

## Abstract

**Background:**

More than 20 million individuals worldwide, especially in the Vidarbha region of India, are affected by sickle cell anemia (SCA), a hereditary condition that results in aberrant hemoglobin S and red blood cell distortion. The condition leads to anemia, organ complications, and recurrent pain crises, making region-specific data necessary for efficient therapy and public health initiatives.

**Objective:**

The goal of the study is to examine the clinical characteristics and unusual manifestations of SCA in the Vidarbha region, with an emphasis on dietary practices, clinical presentations, demographic distribution, and lifestyle factors such as alcohol consumption and smoking.

**Methods:**

This observational cross-sectional study with random sampling will be conducted at Acharya Vinoba Bhave Rural Hospital in Wardha for 3 months. We will recruit 131 individuals aged 18 to 50 years with dominant hemoglobin S and a positive sickling test. A standardized questionnaire addressing clinical symptoms, nutrition, substance use, inheritance patterns, and demographic information will be used to gather data. SPSS (version 17; IBM Corp) will be used for statistical analysis. Data will be summarized using descriptive statistics. Group differences will be evaluated using inferential tests such as 1-way ANOVA, independent 2-tailed *t* tests, and chi-square tests. Associations between symptoms and lifestyle variables will be investigated by correlation analysis. Statistical significance is defined as a 2-tailed *P* value <.05.

**Results:**

The anticipated findings may support the need for targeted regional public health initiatives and underscore the importance of comprehensive screening, detailed patient history, and tailored care strategies for individuals with SCA. As of January 2026, this observational study has not received external funding. Participant recruitment and data collection commenced in January 2026 and are currently ongoing. Data analysis will be undertaken following completion of data collection, and the final results are expected to be submitted for publication in April 2026.

**Conclusions:**

The findings will support the need for focused regional public health initiatives and emphasize the need for thorough screening, patient history, and customized care techniques for SCA.

## Introduction

In 1910, the first clinical case of sickle cell anemia (SCA) in the United States was reported by Dr John Herrick [1]. SCA is a health condition affecting more than 20 million people worldwide [2].

The main cause of SCA is a genetic mutation that affects hemoglobin, the protein in red blood cells that carries oxygen [3]. A point mutation in the *HBB* gene on chromosome 11 is the underlying cause of SCA, as it leads to the production of aberrant hemoglobin, known as hemoglobin S (*HBS*) [4]. The particular mutation involves the replacement of a single nucleotide; namely, thymine replaces adenine. This results in the substitution of valine for glutamic acid at the sixth position of the beta-globin chain. This change alters hemoglobin structure and function, increasing its tendency to polymerize under conditions such as low oxygen tension or dehydration [5]. Due to the autosomal recessive inheritance pattern of SCA [6], an individual needs to inherit 2 copies of the mutated gene, 1 from each parent, to develop the disease. Individuals who inherit 1 mutated gene and 1 normal gene have the sickle cell trait and are usually asymptomatic but can transmit the trait to their offspring [7]. The molecular makeup of HbS is different from that of normal adult hemoglobin A (HBA). Red blood cells deform and have less ability to pass through tiny blood vessels as a result of HbS, which makes them sticky and stiff [8]. Alterations in red blood cell morphology are associated with multiple SCA-related health complications [9]. Polymerization of HbS causes red blood cells to adopt the characteristic sickle shape, which is rigid and prone to hemolysis [10]. These sickled red blood cells clog blood vessels, leading to tissue ischemia, vaso-occlusion, and subsequent organ damage [10].

A significant health burden is caused by SCA, which is characterized by high morbidity and reduced life expectancy, in the Vidarbha region of India [11]. Research and intervention are crucial in this region, including Wardha, because of the high incidence of patients with SCA. The high frequency of SCA in the Vidarbha area enables the study of a large population and the generation of useful region-specific data. The genetic composition of the local population may offer special difficulties and insights into the development and treatment of the disease. The area has unique health care issues, such as restricted access to specialized care; therefore, creating customized health care plans is essential [10].

This study will be conducted to evaluate the observational and clinical profile of patients with SCA attending the Acharya Vinoba Bhave Rural Hospital, Wardha. The findings may underscore the importance of comprehensive screening and detailed patient history in accurately diagnosing and managing the disease. The clinical profiles, characterized by recurrent pain crises, anemia, and potential organ complications, may highlight the need for ongoing monitoring and tailored treatment strategies, with inclusion and exclusion criteria shown in Textbox 1. Observational data may reveal significant patterns in disease prevalence and severity, reinforcing the necessity for targeted public health interventions and continuous support for affected individuals and their families. The purpose of the study is to describe the clinical characteristics and unusual manifestations of SCA and to find associations with lifestyle and demographic factors among people in the Vidarbha area. Specifically, the study aims to examine the age, gender, and ethnic distribution of people with SCA; document and examine their clinical presentations; evaluate their dietary habits; and investigate the connection between clinical symptoms and substance abuse, especially alcohol consumption and tobacco use.

Textbox 1. **Inclusion and exclusion criteria for the study.**

**Inclusion criteria**

- Patients with a positive sickling test and dominant sickle hemoglobin (HbSS)
- Patients in the age group of 18 to 50 years

**Exclusion criteria**

- Age above 50 years
- Other genetic abnormalities
- Individuals with immunocompromising conditions, including HIV infection, organ transplantation, or ongoing chemotherapy.

## Methods

### Overview

**Figure 1.**
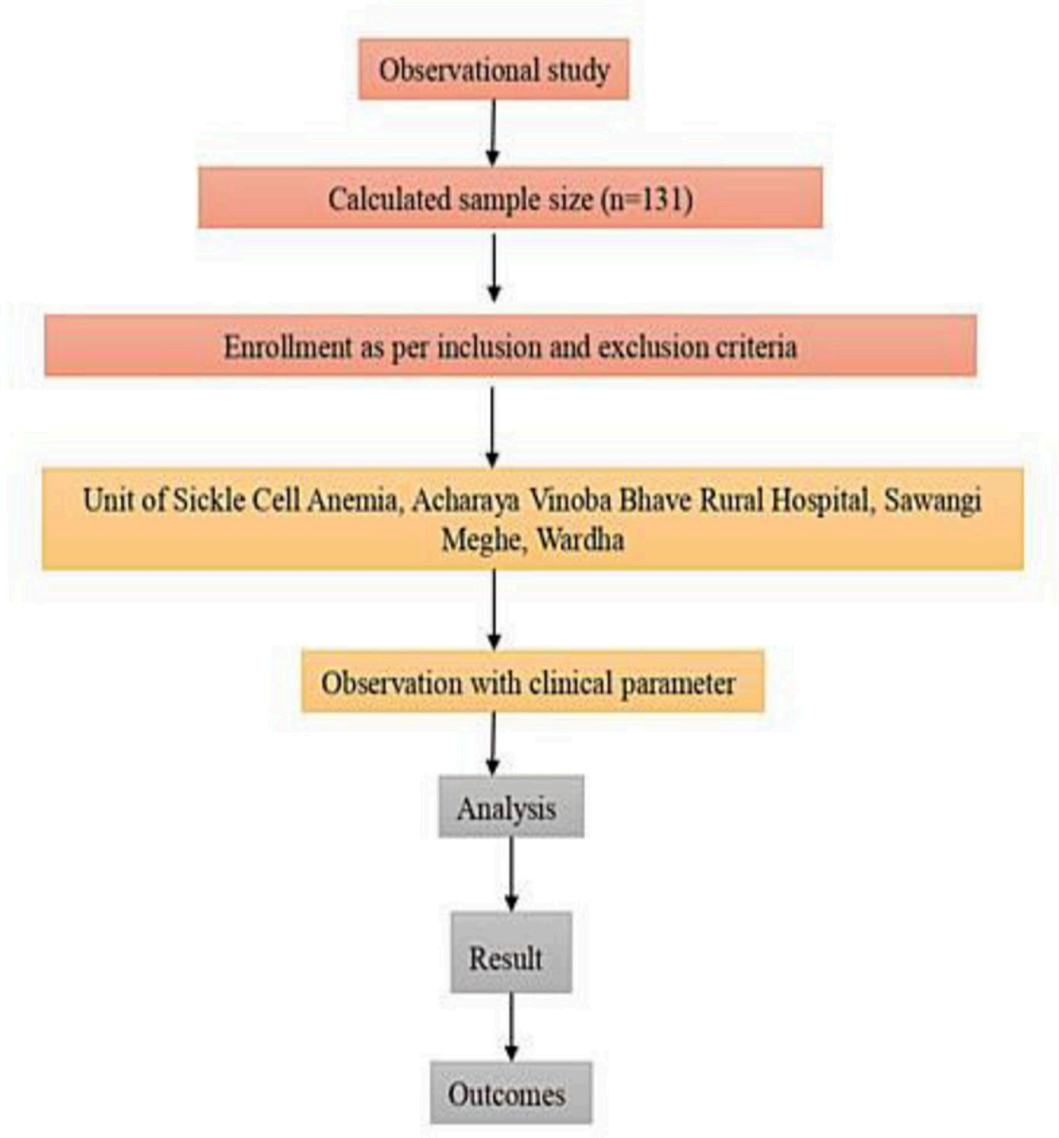
Participant recruitment flowchart (N=131).

This prospective observational cross-sectional study will be conducted for 3 months in the Unit of Sickle Cell Anemia at Acharya Vinoba Bhave Rural Hospital, Datta Meghe Institute of Higher Education and Research, Sawangi, Wardha. Data collection will involve administering a structured questionnaire to gather information on age, gender, area of residence, ethnicity, inheritance patterns, existing symptoms, dietary habits, and history of smoking and alcohol consumption. Objective clinical data, including hemoglobin levels, previous hospitalization records, and documented complications, will be confirmed by reviewing participant hospital case sheets and laboratory reports (as permitted by the consent form), despite the fact that the study does not involve blood or tissue sampling. This approach minimizes the possibility of bias from self-reported responses and ensures data correctness. The collected data will be analyzed to identify rare symptoms of SCA and correlate these findings with various demographic and lifestyle parameters (Figure 1).

### Participants

Participants will be consecutively recruited from the Sickle Cell Outpatient Department of Acharya Vinoba Bhave Rural Hospital for 3 months, using convenience sampling to include all eligible patients aged 18 to 50 years, with the aim of reducing age-related comorbidities. A sample size of 131 was determined based on a regional prevalence of 9.4%, although no separate power analysis for group comparisons was conducted, which is acknowledged as a limitation. Descriptive statistics will summarize demographic and clinical variables, while inferential tests (*t* tests, ANOVA, chi-square test, and correlation analyses) will be used to examine bivariate associations. Multivariable regression analysis will be conducted to adjust for confounders such as age, gender, and lifestyle factors. The inclusion criterion “dominant HbS” will be clarified to differentiate between homozygous (HbSS) and heterozygous (HbAS) genotypes, in line with the autosomal recessive inheritance of SCA.

### Data Collection Procedure

After informed consent has been obtained and the study’s goal has been explained, data will be gathered from patients with SCA who visit the Department of Sickle Cell. Trained interns will administer a structured questionnaire to document demographic and lifestyle factors, such as age, gender, location, ethnicity, inheritance patterns, current symptoms, dietary practices, and history of smoking and alcohol use. The questionnaire’s content validity and clarity were pretested. Cronbach α will be used to evaluate internal consistency (≥0.7 is considered acceptable), and test-retest reliability will be assessed through a 2-week follow-up with approximately 10% of participants. These metrics attest to the tool’s validity and usefulness for clinical and observational sickle cell disease evaluation at the tertiary care facility in Vidarbha. Responses will be documented on paper, and each interview will last for approximately 15 minutes. Records will be stored securely, access will be limited, and they will be disposed of properly after study completion.

### Sample Size

The sample size was determined using the following formula:

n = \frac{Z^{2} \times p \times q}{d^{2}} ]

Where n = required sample size, Z = standard normal deviation at a 95% confidence level (1.96), p = estimated prevalence of the condition, q = 1−p1 - p1−p, and d = allowable error (precision).

### Outcomes

#### Primary Outcome

Assessing the clinical features of patients with SCA will be the main goal of this research, with an emphasis on the frequency and severity of symptoms, including anemia, organ complications, and recurrent pain crises. This outcome will demonstrate the importance of careful screening and a thorough patient history in accurately diagnosing and treating the disease.

#### Secondary Outcome

To support affected individuals and their families, the secondary outcome will involve analyzing observational data to identify significant trends in disease prevalence and severity. This analysis will highlight the need for ongoing monitoring, customized treatment plans, and focused public health initiatives.

### Statistical Methodology

SPSS Statistics for Windows (version 17.0; IBM Corp) will be used for statistical analysis. To summarize demographic and clinical features, descriptive statistics such as mean, SD, median, and frequency distributions will be computed. For inferential analyses, chi-square tests will be used for categorical variables and independent 2-tailed *t* tests or 1-way ANOVA will be used for continuous variables, as applicable. To assess associations between symptoms and nutritional or drug use habits, correlation analyses will be used. 95% CIs will be provided for a 2-tailed *P* value <.05, which is deemed statistically significant. Depending on the amount and type of missing values, missing or incomplete data will be handled using the proper statistical approaches, such as case-wise exclusion or imputation procedures.

### Ethical Considerations

This is an observational study without collection of any type of blood or tissue samples. We received institutional ethics committee approval from Datta Meghe Institute of Higher Education and Research (DMIHER(DU)/IEC/2024/155). Informed consent is sought from all participants, and the participants are informed that they can opt out. All personal information is kept completely confidential, and coded IDs are used to record participant data to guarantee anonymity. To preserve participant anonymity and guarantee adherence to ethical guidelines, all gathered data will be safely kept on password-protected devices that will only be accessible by the study team.

## Results

The study is expected to identify and characterize clinical features of sickle cell anemia, particularly among symptomatic individuals, with attention to severe or uncommon presentations. Recurrent episodes of pain, anemia, and possible organ damage, all suggestive of the disease’s typical vaso-occlusive crises, may be observed. A thorough patient history that will address familial incidence, symptom frequency, and previous medical interventions may prove crucial for making an accurate diagnosis. This all-encompassing strategy will guarantee a complete comprehension and handling of SCA, underscoring the importance of clinical presentation and patient history in successful disease detection and therapy. As of January 2026, this observational study has not received external funding. Participant recruitment and data collection commenced in January 2026 and are currently ongoing. Data analysis will be undertaken following completion of data collection, and the final results are expected to be submitted for publication in April 2026.

## Discussion

### Anticipated Findings

Sickle cell anemia (SCA) affects over 20 million people worldwide [2], and was first described in 1910 by Dr. John Herrick [1]. It is caused by a mutation in the *HBB* gene on chromosome 11, involving a single nucleotide substitution in which thymine replaces adenine, leading to the replacement of glutamic acid with valine at the sixth position of the β-globin chain [4]. This structural alteration produces hemoglobin S (HbS), which polymerizes under conditions of low oxygen tension or dehydration, deforming red blood cells into a sickle shape and causing severe complications. SCA follows an autosomal recessive inheritance pattern; individuals with one mutated gene carry the sickle cell trait and are typically asymptomatic but can transmit the disorder [7]. The disease is highly prevalent in regions of Africa, India, the Middle East, and the Mediterranean [8]. In India, particularly in the Vidarbha region, including Wardha, SCA represents a major public health burden associated with high morbidity and reduced life expectancy [11], making it a critical area for research and intervention. The high prevalence of SCA in Vidarbha provides a substantial population for study and yields meaningful data. The genetic makeup of the population in this region may present unique challenges and insights into the disease’s progression and management. The region faces specific health care challenges, including limited access to specialized care, making it vital to develop tailored health care strategies [10].

Sickle-shaped red blood cells can cause a range of problems. They can get trapped in small blood vessels, blocking the flow of blood and causing pain and organ damage. Normal red blood cells survive for about 120 days, whereas sickled cells only last about 10 to 20 days. This leads to a chronic shortage of red blood cells, known as anemia [12]. Blocked blood flow can cause episodes of severe pain, known as pain crises or vaso-occlusive crises. These can occur without warning and may require hospitalization. People with SCA have a higher risk of infections because sickle cells can damage the spleen, an organ that helps fight infections. Some common clinical manifestations include fatigue, weakness, and shortness of breath due to a lack of healthy red blood cells; sudden, severe pain in different parts of the body, often in the bones, chest, and joints; and painful swelling, known as dactylitis, is often the first symptom in infants. Children with SCA may experience delayed growth compared with their peers and attain puberty at an older age. Sickle cells can block the tiny blood vessels that supply the eyes, leading to vision issues. Blocked blood flow to the brain can cause a stroke, a serious complication of SCA. Red blood cells with aberrant HbS change in morphology, becoming sticky and rigid, which can cause a number of health issues. The characteristic sickle-shaped deformation of red blood cells is associated with hemolysis and occlusion of small vessels, leading to tissue ischemia, organ damage, and vaso-occlusion [8]. Although there is no universal cure for SCA, several treatments can help manage symptoms and reduce complications. Pain relief is a primary treatment goal. Medications range from over-the-counter pain relievers to stronger prescription drugs. Folic acid (1 mg) and hydroxyurea (500 mg) help reduce the frequency of pain crises and the need for blood transfusions. It works by increasing the production of fetal hemoglobin, which prevents sickling of red blood cells [13]. Regular blood transfusions can help reduce the risk of stroke and other complications by increasing the proportion of normal red blood cells [14]. While hematopoietic stem cell transplantation offers the only potential for cure, its implementation is limited by the high risk of serious complications. This is currently the only potential cure for SCA. It involves replacing the patient’s bone marrow with healthy marrow from a donor, but it is only suitable for some patients due to the risks involved. Comprehensive preventive care, including vaccination, prophylactic antibiotics, maintenance of adequate hydration, avoidance of extreme temperatures, and routine medical monitoring, is fundamental to improving patient outcomes [15]. Some promising areas of research for SCA include gene therapy approaches, new medications, and genome editing technologies, such as CRISPR-Cas9 [16]. The pathophysiological mechanisms underlying the clinical manifestations of SCA underscore the need for thorough screening, customized care, and ongoing monitoring to effectively manage the disease [17].

### Limitations

It is important to acknowledge the limitations of this study. Recruiting individuals from a single institution may limit the generalizability of the findings, and the short study duration may make it more difficult to discover long-term effects. Furthermore, there may be selection bias because the research sample may not be representative of the entire eligible population. Recall bias resulting from self-reported eating habits may potentially impact the quality and reliability of the dietary data. These considerations should be taken into account by researchers when assessing the results. Furthermore, there is a chance of recall or reporting bias because the majority of the data collection will be done through self-reported questionnaires; although, attempts will be made to cross-check participant medical records and laboratory data to improve reliability and reduce the possibility of clinical outcomes being misclassified.

### Conclusions

This investigation may offer crucial insights into the clinical spectrum of SCA and underscore the necessity for customized interventions in the Vidarbha region. The findings highlight the importance of comprehensive screening and a detailed patient history in accurately diagnosing and managing the disease. The clinical profiles, characterized by recurrent pain crises, anemia, and potential organ complications, emphasize the need for ongoing monitoring and tailored treatment strategies. Observational data may reveal significant patterns in disease prevalence and severity, reinforcing the necessity for targeted public health interventions and continuous support for affected individuals and their families.

## Abbreviations

HbA: hemoglobin A
HbS: hemoglobin S
SCA: sickle cell anemia
